# Anti-PD-1 Therapy Response Predicted by the Combination of Exosomal PD-L1 and CD28

**DOI:** 10.3389/fonc.2020.00760

**Published:** 2020-05-27

**Authors:** Chaoxu Zhang, Yibo Fan, Xiaofang Che, Min Zhang, Zhi Li, Ce Li, Shuo Wang, Ti Wen, Kezuo Hou, Xinye Shao, Yunpeng Liu, Xiujuan Qu

**Affiliations:** ^1^Department of Medical Oncology, The First Hospital of China Medical University, Shenyang, China; ^2^Key Laboratory of Anticancer Drugs and Biotherapy of Liaoning Province, The First Hospital of China Medical University, Shenyang, China

**Keywords:** exosomal PD-L1, CD28, immune checkpoint inhibitor, response prediction, anti-PD-1 therapy

## Abstract

Anti-PD-1 therapy has been approved for cancer treatment. However, the response rate is unsatisfactory. The expression of PD-L1 in tumor tissues is unreliable to predict the treatment response. Recent studies have suggested that exosomal PD-L1 not only exerts immunosuppressive effects but also plays a significant role in the development of tumor microenvironment. Thus, the present study aims to investigate exosomal PD-L1 in improving its predictive value and efficacy. A total of 44 patients of advanced tumors of several types, treated with anti-PD-1 therapy, were enrolled. Exosomes were collected and purified from plasma. The exosomal PD-L1 was detected with ELISA. The cytokines were measured with the MILLIPLEX magnetic bead assay. Compared to the responders, exosomal PD-L1 of the non-responders was significantly higher than that of the responders (*P* = 0.010) before the treatment. Concurrently, exosomal PD-L1 and tumor burden decreased when the therapy was effective. And, the baseline expression of CD28 was higher in the responders than that in the non-responders (*P* = 0.005). Univariate and multivariate analyses validated with 1,000 times bootstrapping suggested that high exosomal PD-L1 and low CD28 expressions were negative factors for progression-free survival (PFS) of the patients who underwent anti-PD-1 treatment. The combination of exosomal PD-L1 and CD28 obtained more area under the curve (AUC) of receiver operating characteristic (ROC) (AUC 0.850 vs. 0.784 vs. 0.678) and showed a higher probability of no progression via nomograph. These findings suggested that the expression of exosomal PD-L1 and CD28 could serve as the predictive biomarkers for clinical responses to anti-PD-1 treatment.

## Introduction

Nowadays, immunotherapy has become the focus and innovation in anti-tumor therapy (1–3). The interaction between programmed cell death-1 (PD-1) and its ligand (PD-L1) exerts a considerable effect on immune escape, tumor progression, and metastasis (4). In recent years, various types of immunotherapy, such as immune checkpoint blockade, have been used in numerous clinical trials and approved for the treatment, irrespective of the tumor types (5, 6). PD-1 monoclonal antibody, an immune checkpoint blockade, induces an antitumor immune response by blocking the connection between PD-1 and PD-L1. Overall, the effective rate of monotherapy of the PD-1 antibody is only 10–25% (7, 8). Therefore, finding the novel biomarkers for predicting the efficacy of anti-PD-1 treatment is an urgent requisite.

Tumor mutation burden (TMB) is a potential biomarker defined as the total number of somatic mutations per megabase or non-synonymous mutations in the tumor tissues, including substitutions and insertion deletions (9). In some trials, the objective response rate was high for PD-1/PD-L1 inhibitors in patients with high TMB (10–12). In addition, Mismatch repair deficient (dMMR) leads to the accumulation of mismatched bases in DNA replication process, which in turn, causes microsatellite instability (MSI). Tumors with dMMR or MSI-H are the major components of high TMB tumors (13). The high TMB tumors harbor a large number of new antigen loads and tumor immunogenicity (14). Therefore, MSI-H/dMMR or high TMB are considered as the potential biomarkers for predicting the efficacy of anti-PD-1 treatment (14, 15). However, the limitation of MSI-H and high TMB population, the inconsistency of TMB detection platform, the uncertainty of cutoff values, the heterogeneity and difficulty in detecting specimens limit the application and development of this treatment (14).

As the key factor on the target pathway, the expression of PD-L1 on tumor cells detected by immunohistochemistry (IHC) has been deemed as a potential biomarker which responds to PD-1 monoclonal antibody (8, 9, 14, 16). Several studies reported that patients with high PD-L1 expression might benefit from the treatment, while other researches showed that the outcome of patients with low or no PD-L1 did not differ from the high PD-L1 cohort (17–19). The cutoff value of PD-L1 was 1–50%, as deduced from various clinical trials (20). Moreover, the existence of membranous PD-L1 is unstable and the acquisition is inconvenient, which makes the efficiency of PD-L1 expression for the prediction of the treatment response unreliable (14).

Recent studies have revealed several types of PD-L1, such as soluble, exosomal, and membranous PD-L1 (21, 22). Exosomes, carrying a variety of biological information such as DNA, RNA, and protein, are extracted and purified from the plasma of patients with various tumors (23). Exosomal PD-L1 is more stable than the soluble type and more easily available as compared to the membranous type. Interestingly, it is not confined to local tumors but exerts its immunosuppressive function in distant areas. Recently, several studies showed that exosomal PD-L1 had an immunosuppressive effect, including the inhibition of T cell activation, promotion of T cell apoptosis, suppression of immune memory, and promotion of the tumor growth (22–26). The application of the exosome inhibitor enhanced the efficacy of the PD-1 monoclonal antibody (26). Another study demonstrated that melanoma patients with high exosomal PD-L1 baseline expression exhibited a poor response to anti-PD-1 antibodies (24).

Due to the complexity of the immune response, a single indicator does not fully predict the efficacy of immunotherapy. In addition to the inhibition of the immune microenvironment of tumor cells represented by PD-L1, the exhausted state of T cells indicated by PD-1, LAG-3, and Tim-3, the activation state of T cells marked by CD8 and CD28, and the T cell killing function effectuated by INF-γ, granzyme B, and perforin also provide a vital and predictive effect on the efficacy of immunotherapy. Therefore, whether the multi-index combination can predict the response of multiple types of tumors to anti-PD-1 therapy is yet to be elucidated.

## Materials and Methods

### Patients and Samples Collection

A total of 44 patients were enrolled between June 2017 and April 2019 with Stage IV pan-cancer (Table 1), including 14 lung adenocarcinoma, 10 lung squamous carcinoma, 5 esophageal carcinoma, 2 colorectal carcinoma, 2 cholangiocarcinoma, 2 nasopharyngeal carcinoma, 2 lung small cell carcinoma, 1 lung large cell carcinoma, 1 gastric adenocarcinoma, 1 tongue squamous cell carcinoma, 1 duodenal adenocarcinoma, 1 renal cell carcinoma, 1 hepatocellular carcinoma, and 1 malignant melanoma. Patients were enrolled from age 18 to 80 with histologically confirmed carcinoma. Further eligibility criteria were an Eastern Cooperative Oncology Group (ECOG) performance status of 0 or 1, adequate hematological and biochemical values, and no known hypersensitivity to PD-1 monoclonal antibody. Patients who had previously been treated with an agent targeting immune checkpoint pathways (including those targeting PD-1, PD-L1 or PD-L2, or CTLA-4) were excluded. All the patients from The First Affiliated Hospital of China Medical University were treated by anti-PD-1 therapy according to the standard regimens. Clinical information of all patients was retrieved from the Hospital Information System. Clinical response was determined as best response based on immune-related RECIST (irRECIST). Progression-free survival (PFS) was calculated from the time of treatment till progression or the last follow-up visit (August, 2019).

**Table 1:** Clinical characteristics of patient.

**Variables**	**Patients** **(*n* = 44)**	**Responders** **(*n* = 20)**	**Non-responders** **(*n* = 24)**
**Age**			
Median	59	60	57.5
Range	29–76	29–74	30–76
**Gender**			
Male	31 (70.45%)	16 (80.00%)	15 (62.50%)
Female	13 (29.55%)	4 (20.00%)	9 (37.50%)
**Number of previous treatment lines**			
0–1	36 (81.82%)	16 (80.00%)	20 (83.33%)
≥2	8 (18.18%)	4 (20.00%)	4 (16.67%)
**Response of previous treatment lines**			
Response	10 (22.73%)	3 (15.00%)	7 (29.17%)
Non-response	25 (56.82%)	8 (40.00%)	17 (70.83%)
NA or NR	9 (20.45%)	9 (45.00%)	0
**Type of metastatic sites**			
Lymph nodes	29 (65.91%)	15 (75.00%)	14 (58.33%)
Bone	11 (25.00%)	4 (20.00%)	7 (29.17%)
Liver	8 (18.18%)	4 (20.00%)	4 (16.67%)
Lung	6 (13.64%)	3 (15.00%)	3 (12.50%)
Pleura	6 (13.64%)	2 (10.00%)	4 (16.67%)
Others	12 (27.27%)	6 (30.00%)	6 (25.00%)
**Number of metastatic lesions**			
1	18 (40.91%)	7 (35.00%)	11 (45.83%)
≥2	26 (59.09%)	13 (65.00%)	13 (54.17%)
**Cancer type**			
Lung adenocarcinoma	14 (31.82%)	3 (15.00%)	11 (45.83%)
Lung squamous cell carcinoma	10 (22.73%)	6 (30.00%)	4 (16.67%)
Esophageal carcinoma	5 (11.36%)	2 (10.00%)	3 (12.50%)
Colorectal carcinoma	2 (4.55%)	1 (5.00%)	1 (4.17%)
Cholangiocarcinoma	2 (4.55%)	1 (5.00%)	1 (4.17%)
Nasopharyngeal carcinoma	2 (4.55%)	1 (5.00%)	1 (4.17%)
Lung small cell carcinoma	2 (4.55%)	0	2 (8.33%)
Lung large cell carcinoma	1 (2.27%)	1 (5.00%)	0
Gastric adenocarcinoma	1 (2.27%)	1 (5.00%)	0
Tongue squamous cell carcinoma	1 (2.27%)	1 (5.00%)	0
Duodenal adenocarcinoma	1 (2.27%)	0	1 (4.17%)
Renal cell carcinoma	1 (2.27%)	1 (5.00%)	0
Hepatocellular carcinoma	1 (2.27%)	1 (5.00%)	0
Malignant melanoma	1 (2.27%)	1 (5.00%)	0

Plasma samples from patients were collected before treatment and accompanied with efficacy evaluation under the informed consent of patients. We collected whole blood in Venous Blood Collection Tubes containing EDTA, centrifuged blood samples in primary blood collection tubes for 10 min at 3,000 rpm and 4°C using a swinging bucket rotor within 1 h, and carefully transferred the upper plasma phase to a new tube and stored it in −80°C refrigerator.

### Ethics Approval

This study was carried out in accordance with the recommendations of Scientific Ethics NO. 2019-142-2, China Medical University. The protocol was approved by the Ethics Committee of China Medical University. All subjects gave written informed consent in accordance with the Declaration of Helsinki.

### Purification of Exosomes

Exosome was collected and purified from plasma with centrifugation and exosome isolation kit (System Biosciences, Catalog #EXOQ5A-1) following the standard protocol strictly (27). Cell-free plasma was first centrifugated for 15 min at 4,500 g and 4°C to remove the cellular fragments and cell debris. Then, exosomes were purified from the supernatants with the exosome isolation kit. The final pellet containing exosomes were re-suspended in the same volume of PBS as the plasma as they were originally derived from. Exosomes for TEM, ELISA, flow cytometry analysis (FACS), and Western Blot were resuspended in PBS. Exosomes sample were diluted 100 times in PBS for NanoSight LM10 (NanoSight Ltd) analysis.

### Western Blot Analysis

Exosomal marker proteins, CD9 and CD63, and PD-L1 on exosomes were measured from exosome suspension by western blotting according to previous research (28). Antibodies against CD9, CD63, and PD-L1 were from Cell Signaling Technology (Danvers, MA).

### Electron Microscopy

Exosomes from patients' plasma were fixed at optimal concentration and settled on a 400-mesh carbon/formvar covered grids. They were supposed to absorb the formvar for at least 1 min. The samples were viewed with the Tecnai Bio Twin transmission electron microscope (FEI) and images were obtained with an AMT CCD Camera (Advanced Microscopy Techniques).

### Flow Cytometry

Exosomes were resuspended in 100 ul PBS and then added 10 ul aldehyde/sulfate beads (Invitrogen, Catalog #A37304) into it, rotated at room temperature for 15 min. We added 600 ul PBS and rotated at 4°C overnight and 400 ul 1 M glycine rotated at room temperature for 30 min. Samples were spined at 12,000 rpm for 1.5 min and aspirated supernatant, resuspended precipitate in 100 ul 10% BSA, rotating at room temperature for 45 min. Specimens staining was performed for 30 min at 4°C with the following antibodies: PE Anti-Human-PD-L1 (BioLegend, Catalog #329706), Alexa Fluor® 647 anti-human CD279 (PD-1) Antibody (BioLegend, Catalog # 329910), PerCP/Cyanine 5.5 anti-human CD9 Antibody (BioLegend, Catalog #312110) and FITC anti-human CD63(BioLegend, Catalog #329906) tested in Beckman Coulter CytoFLEX (CA, USA).

### ELISA

The expression of exsomal PD-L1 and exosomal PD-1 from patients' plasma was detected by enzyme-linked immunosorbent assay (ELISA) with the human PD-L1/B7-H1 DuoSet ELISA kit (R&D, Catalog # DY156). We coated 96 well microplates (R&D, Catalog # DY990) with Human PD-L1 Capture Antibody overnight at 4°C and two hundred microliters of blocking buffer was added for 1 h incubation at normal temperature. One hundred microliters of exosome suspension or plasma or standard were added to each well for 2 h at room temperature. Then each well was washed for four times with wash buffer, and 100 μL of human PD-L1 Detection Antibody was added to each well for 2 h with coverage. Microplates were washed for four times with wash buffer and 100 μL of Streptavidin conjugated to horseradish-peroxidase diluted in blocking buffer was then added to each well. After 20 min incubation at dark room, 100 μL Substrate Solution which combined by equivalent Color Reagent A (H_2_O_2_) and Color Reagent B (Tetramethylbenzidine) (R&D, Catalog # DY999) was added to each well and incubated for 20 min at dark room. Then, 50 μL of stop solution was added to each well, and results were then measured immediately by Bio-RADi Mark (Bio-RAD laboratories Inc., Kyoto Japan) with absorbance at 450 nm.

### Detection of Several Cytokines

The MILLIPLEX magnetic bead assay (29) was used as per manufacturer's instructions (Millipore) to measure cytokines secretion from activated CD8+ T cells and Co-inhibitory or co-stimulatory factors of Immune Checkpoint protein via patients' serum. Seventeen cytokines were detected by Human CD8+ T-Cell Magnetic Bead Panel (Cat.# HCD8MAG-15K), including GM-CSF, sCD137, IFN-ɤ, IL-2, IL-4, IL-5, IL-6, IL-10, IL-13, sFas, sFasL, Granzyme A, Granzyme B, MIP-1α, MIP-1β, TNF-α, and Perforin. 96-well plates containing assay buffer diluted at a 1:1 ratio were loaded with 25 μL of samples, standards, and controls. Magnetic beads coated with antibodies were added to each well before the plate was sealed and incubated overnight at 4°C with shaking. Then, the plate was washed twice and biotinylated detection antibodies were added before it was sealed again and incubated at room temperature with rocking for 1 h. Streptavidin-PE was then added and the plate was rocked at room temperature for another 30 min. The plate was washed twice again and loaded with sheath fluid to be read on the MAGPIX system. The Milliplex Analyst software was used as per manufacturer's instructions (Luminex, Austin, TX) for data analysis. In addition, several Co-inhibitory factors (BTLA, TIM-3, LAG-3, CTLA-4) and Co-stimulatory factors (CD27, CD28, CD40, HVEM, TLR-2, GITR, GITRL, ICOS, CD80, CD86) were examined by Human Immuno-Oncology Checkpoint Protein Panel (Catalog # HCKPMAG-11K).

### Statistical Analysis

The difference expression of indexes between responder and non-responder were compared by the unpaired Student's *t*-test for continuous variables. Clinicopathological parameters were divided by median and transformed to categorical variable. Exosomal PD-L1, exosomal PD-1 and immune-related factors were divided by optimal cutoff, which was determined by X-tile software. Kaplan–Meier curves were used for PFS analysis. Univariate and multivariable associations between PFS and exosomal PD-L1, CD28 as well as other clinicopathological parameters were measured by Cox PH regression model, with hazard ratios (HR) and 95% confidence interval (CI). The validation of the Cox model was checked with the 95% CI using bootstrapping (1,000 replications). To evaluate the prediction accuracy of exosomal PD-L1 and CD28, receiver operating characteristic (ROC) curves and resulting area under the curve (AUC) were constructed.

All statistical tests were two-tailed with a *P* < 0.05 considered significant. IBM SPSS Statistics 21 and R software (version 3.1.1) was used for the above statistical analysis.

## Results

### Soluble PD-L1 and PD-1 Cannot Predict the Response of Anti-PD-1 Therapy

**Figure 1:**
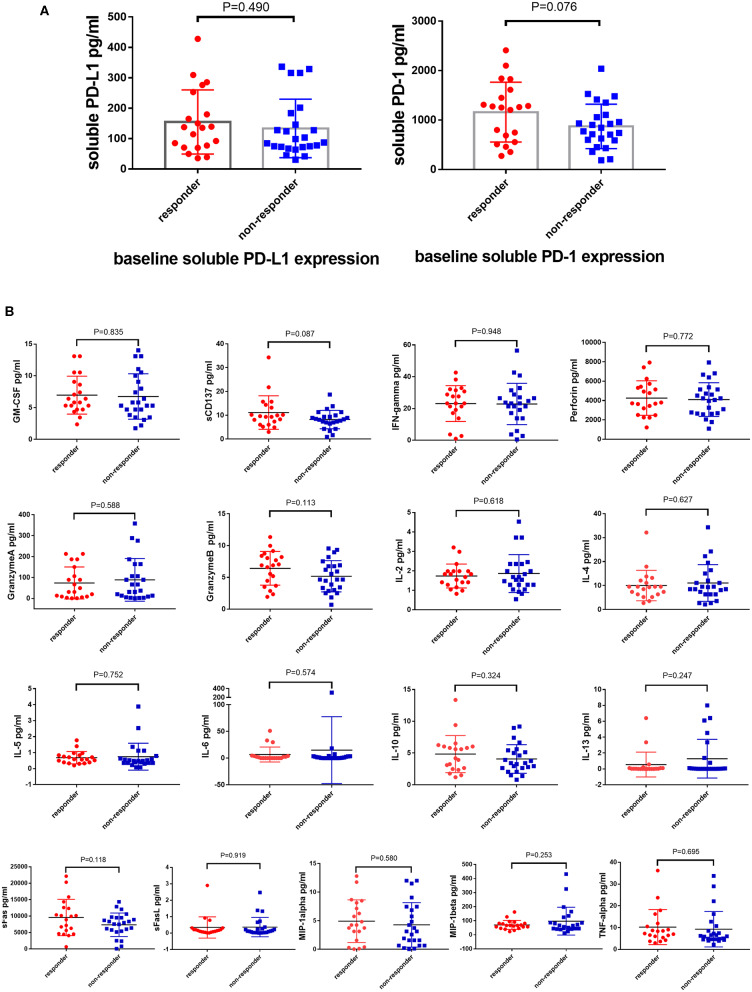
Soluble PD-L1, PD-1, and T cells related cytokines cannot predict the response of anti-PD-1 therapy. Difference expression of soluble PD-L1, PD-1 **(A)** and T cells related cytokines **(B)** from 100 μL serum between responders (*N* = 20) and non-responders (*N* = 24) underwent anti-PD-1 monotherapy compared by the Unpaired Student's *t*-test. *P*-values less than 0.05 was considered that there existed statistical differences.

The expression of soluble PD-L1 and PD-1 was detected in the patient plasma before the treatment of PD-1 monoclonal antibody and was found to be slightly higher in the responder than the non-responder cohort with no significance (*P* = 0.490 and *P* = 0.076, Figure 1A).

### T Lymphocyte-associated Cytokines Cannot Predict the Response of PD-1 Inhibitors

The anti-tumor effect of PD-1 inhibitors is based on the activity and capacity of T lymphocytes. Therefore, the baselines level of T lymphocytes activity related cytokines, such as GM-CSF, sCD137, IFN-ɤ, IL-2, IL-4, IL-5, IL-6, IL-10, IL-13, sFas, sFasL, Granzyme A, Granzyme B, MIP-1α, MIP-1β, TNF-α, and Perforin, was detected using the MILLIPLEX magnetic bead assay. However, the expression of these cytokines did not differ between the responder and non-responder cohorts (Figure 1B).

### High Expression of Exosomal PD-L1 and PD-1 Suggested Poor Efficacy Before Anti-PD-1 Treatment

**Figure 2:**
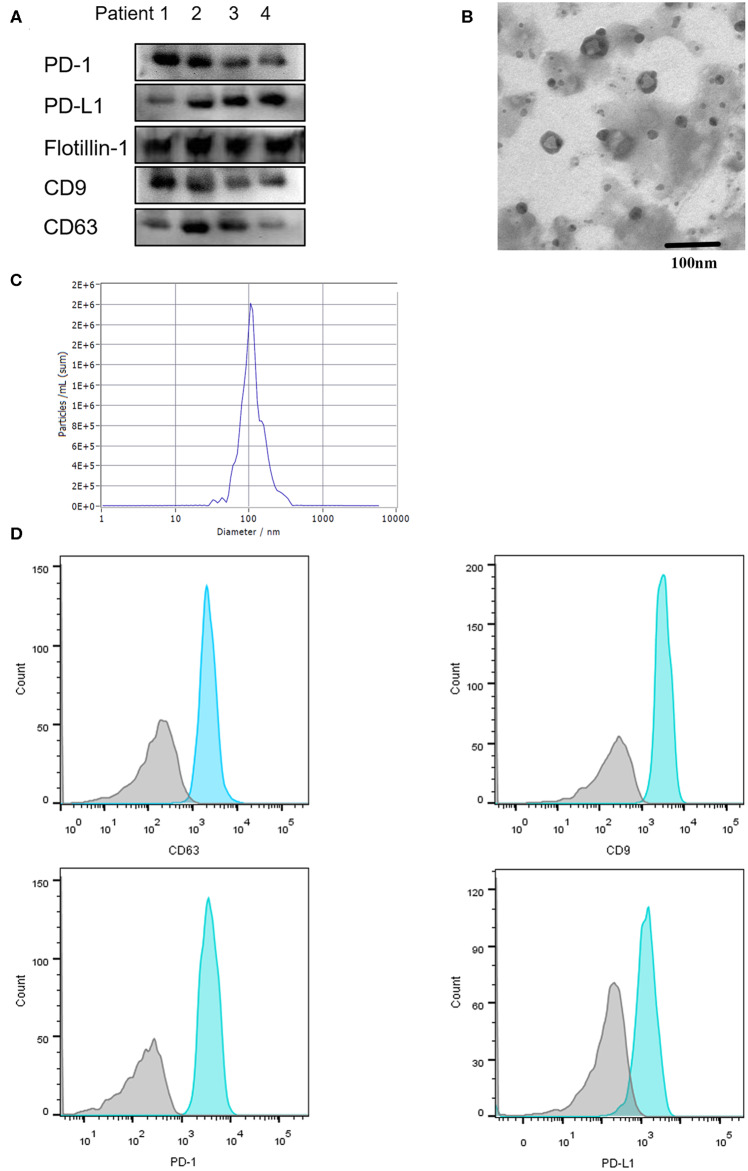
Characterization of serum-derived exosomes. Exosomes were purified from 100 μL serum. **(A)** Exosomal protein CD9, CD63, Flottin-1 and the expression of PD-1 and PD-L1 on exosomes were verified by western blotting. **(B)** Exosomes isolated from serum were observed under electron microscopy (TEM) with 50–150 nm in diameter. Scale bar: 100 nm. **(C)** Concentration and size distribution of exosomes were analyzed by NanoSight. **(D)** Flow Cytometry was performed for the exosomes surface protein CD9, CD63 and exosomal PD-1, exosomal PD-L1 detection.

**Figure 3:**
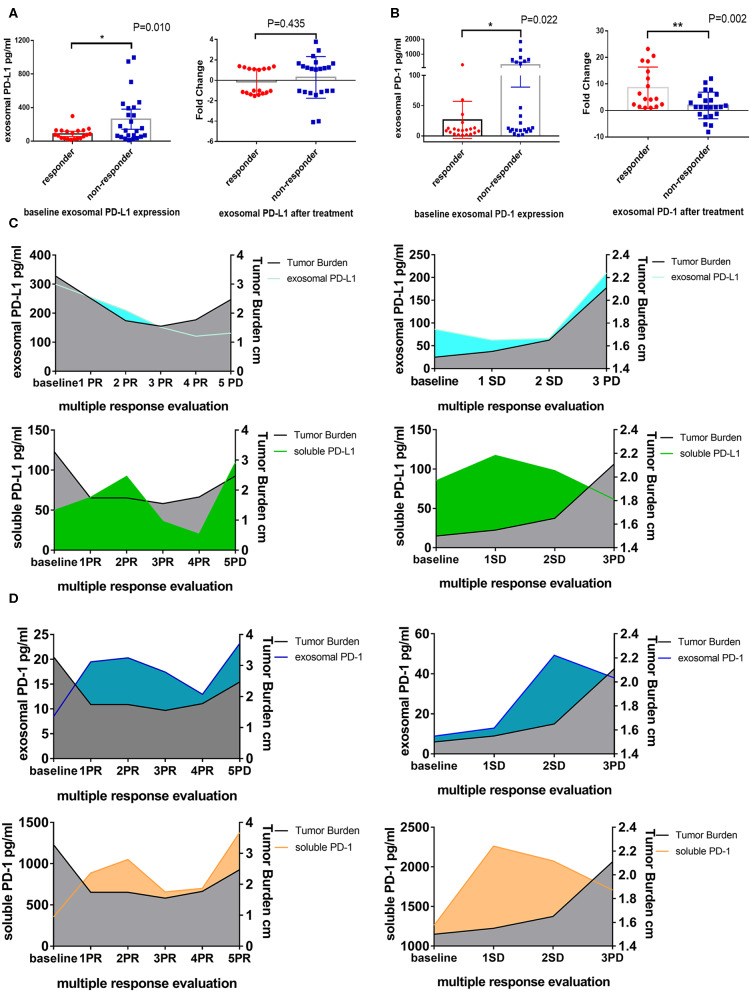
Difference expression of exosomal PD-L1 and PD-1 in responders and non-responders. **(A)** Plot of circulating exosomal PD-L1 levels at baseline and fold-change after anti-PD-1 treatment in responders (*N* = 20) and non-responders (*N* = 24). **(B)** Plot of circulating exosomal PD-1 levels at baseline and fold-change after anti-PD-1 treatment in responders (*N* = 20) and non-responders (*N* = 24). The two-tailed Unpaired Student's *t*-test was used in statistical analysis where appropriate to evaluate the statistical significance (*P < 0.05, **P < 0.01). **(C)** Dynamic change between exosomal PD-L1, soluble PD-L1 and treatment response in two typical patients. With the response of anti-PD-1 treatment, the tumor burden and exosomal PD-L1 but not soluble PD-L1 decreased. When the progression of disease, the tumor burden and exosomal PD-L1 increased. **(D)** Dynamic change between exosomal PD-1, soluble PD-1 and treatment response in two typical patients. Exosomal PD-1 was increased after anti-PD-1 therapy in nearly all patients. With the decline of the tumor burden, exosomal PD-1 was decreased. The change of soluble PD-1 was irregular.

Exosomes are extracellular vesicles secreted from several different types of cells, especially cancer cells, which contain integrated nucleic acids and various proteins. Exosomal PD-L1 was proved to be a vital element of the immune microenvironment of the tumor. In our study, exosomes were confirmed to exist in the plasma by Western blotting, electron microscope, Nanosight and flow cytometry (Figures 2A–D), moreover, PD-1 and PD-L1 were detected on exosomes by Western blotting and flow cytometry (Figures 2A,D). Next, we enriched and purified the exosomes and then tested the level of exosomal PD-1 and PD-L1. Compared to the responders, exosomal PD-L1 of non-responders was remarkably higher (*P* = 0.010) before anti-PD-1 therapy (Figure 3A). After therapy, the fold-change in exosomal PD-L1 decreased in the responder cohort but increased in the non-responder cohort without significant difference (*P* = 0.435, Figure 3A). Surprisingly, the expression of exosomal PD-1 also differed significantly between the two groups. Before undergoing treatment, a lower exosomal PD-1 was detected in the responders than non-responders (*P* = 0.022, Figure 3B). Moreover, exosomal PD-1 increased after treatment in a majority of patients irrespective of the response. The fold-increase in the level of exosomal PD-1 was much higher in the responder cohort than in the non-responders (*P* = 0.002, Figure 3B). Along with efficacy evaluation, exosomal PD-1 and PD-L1 were measured dynamically, and the expression was found to correspond with the curative effect and tumor burden (Figures 3C,D).

### High Level of Immunity Factors at Baseline Indicated a Favorable Effect of PD-1 Inhibitors

**Figure 4:**
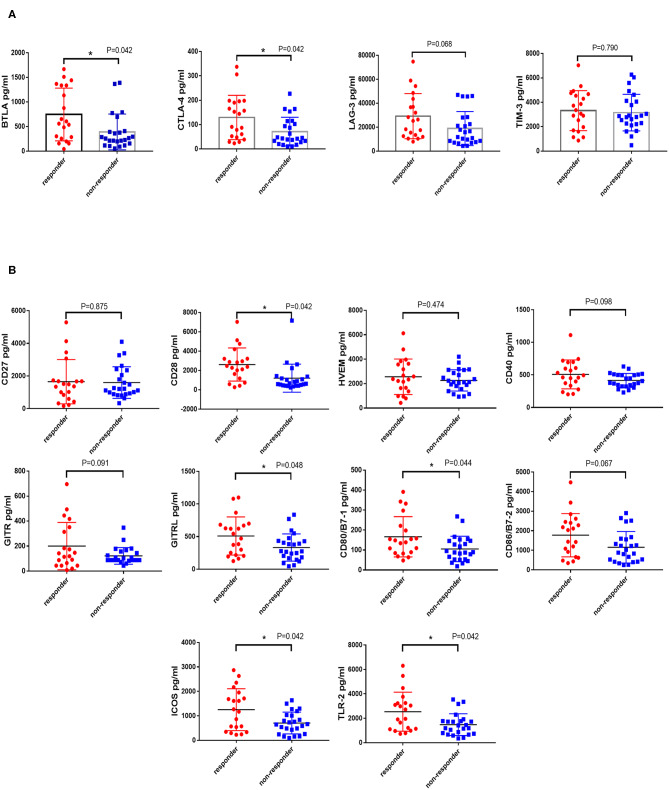
Difference expression of co-inhibitory and co-stimulatory factors in responders and non-responders. The levels of four co-inhibitory factors (BTLA, TIM-3, LAG-3, and CTLA-4) and several co-stimulatory factors (CD27, CD28, CD40, HVEM, TLR-2, GITR, GITRL, ICOS, CD80, and CD86) on patients' serum were measured by the MILLIPLEX magnetic bead assay. Characterization of co-inhibitory **(A)** and co-stimulatory factors **(B)** expression in patients who responded or non-responded to PD-1 inhibitors were compared by the Unpaired Student's *t*-test. *P*-values less than 0.05 was considered that there existed statistical differences (*P < 0.05). *P*-values were corrected by FDR (False discovery rate) of Multiple Comparisons Correction.

Co-inhibitory and co-stimulatory factors react to the ability of antitumor immunity. Therefore, we measured the level of four co-inhibitory factors, such as BTLA, TIM-3, LAG-3, and CTLA-4, and several co-stimulatory factors on patients' serum, including CD27, CD28, CD40, HVEM, TLR-2, GITR, GITRL, ICOS, CD80, and CD86. We revealed that the expression of BTLA, LAG-3, and CTLA-4 in the responders was higher than non-responders (P_BTLA_ = 0.013, P_LAG−3_ = 0.044, P_CTLA−4_ = 0.015, Figure S1A). In addition, the level of CD28, CD80, CD86, GITRL, ICOS, and TLR-2 in the responders was significantly higher than non-responders (P_CD28_ = 0.005, P_CD80_ = 0.019, P_CD86_ = 0.038, P_GITRL_ = 0.024, P_ICOS_ = 0.009, P_TLR−2_ = 0.008, Figure S1B). In consideration of the plenty of analytes, FDR (False discovery rate) of Multiple Comparisons Correction was implemented and the *P*-value of BTLA, CTLA-4, LAG-3, and TIM-3 was 0.042, 0.042, 0.068, and 0.790 (Figure 4A). The corrected *P*-value of co-stimulatory factors was illustrated in Figure 4B (P_CD27_ = 0.875, P_CD28_ = 0.042, P_HVEM_ = 0.474, P_CD40_ = 0.098, P_GITR_ = 0.091, P_GITRL_ = 0.048, P_CD80_ = 0.044, P_CD86_ = 0.067, P_ICOS_ = 0.042, P_TLR−2_ = 0.042).

**Figure 5:**
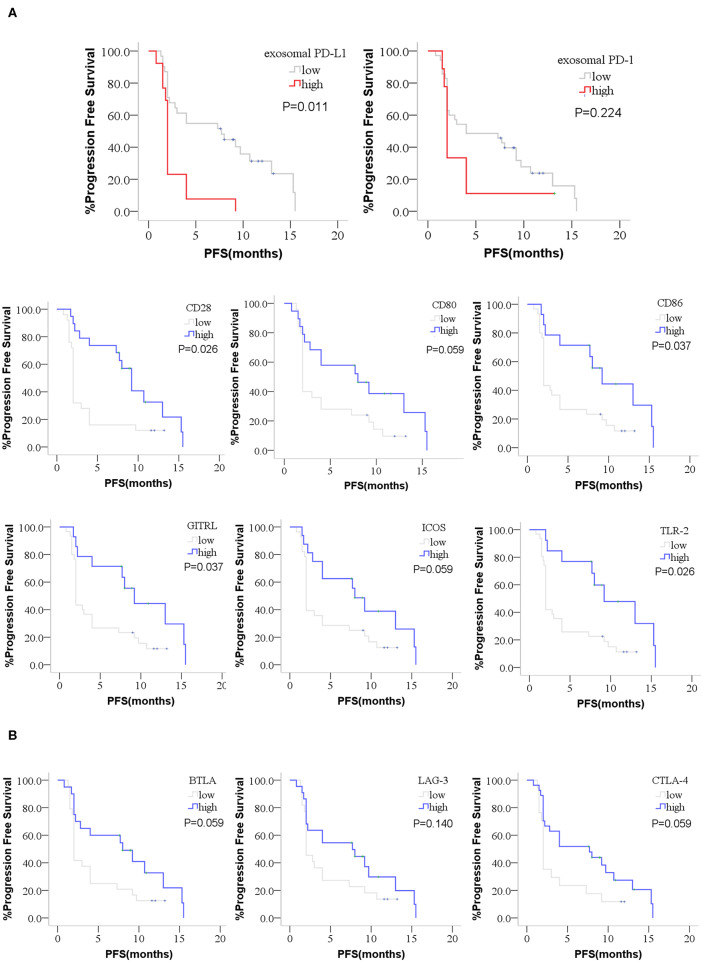
Kaplan-Meier curves for PFS of anti-PD-1 treatment. The difference PFS of anti-PD-1 treatment between high or low group of exosomal PD-L1, exosomal PD-1, co-stimulatory factors and co-inhibitory factors were performed by Kaplan-Meier curves. **(A)** High CD28, CD80, CD86, GITRL, ICOS, TLR-2, and low exosomal PD-L1 patients showed a prolonged PFS after the anti-PD-1 treatment. The prolonged PFS in the low exosomal PD-1 patients was not statistical. **(B)** High BTLA and CTLA-4 showed a prolonged PFS. The prolonged PFS in the high LAG-3 patients was not statistical. *P*-values less than 0.05 was considered that there existed statistical differences. *P*-values were corrected by FDR (False discovery rate) of Multiple Comparisons Correction.

The optimal cutoff value of these factors was obtained by X-tile software. The cutoff point for exosomal PD-1 was determined as 297.8 pg/mL, that for exosomal PD-L1 was 149.0 pg/mL, 1,420 pg/mL for CD28, 130 pg/mL for CD80, 1,674 pg/mL for CD86, 475.1 pg/mL for GITRL, 1,036 pg/mL for ICOS, 2,289 pg/mL for TLR-2, 408.5 pg/mL for BTLA, 17,896 pg/mL for LAG-3, and 48.2 pg/mL for CTLA-4. According to the optimal cutoff point, high expression of exosomal PD-L1, exosomal PD-1, CD28, CD80, CD86, GITRL, ICOS, TLR-2, BTLA, LAG-3, and CTLA-4 was detected in 13 (29.5%), 9 (20.5%), 19 (43.2%), 19 (43.2%), 14 (31.8%), 14 (31.8%), 16 (36.4%), 13 (29.5%), 20 (45.5%), 22 (50.0%), and 27 (61.4%) patients, respectively. Furthermore, high CD28, CD80, CD86, GITRL, ICOS, TLR-2, BTLA, CTLA-4 and low exosomal PD-L1 cohorts showed a significantly prolonged PFS after the treatment (median_CD28_, 2 vs. 9.2 months, *P* = 0.005; median_CD80_, 2 vs. 8 months, *P* = 0.048; median_CD86_, 2 vs. 9.2 months, *P* = 0.017; median_GITRL_, 2 vs. 9.2 months, *P* = 0.017; median_ICOS_, 2 vs. 8 months, *P* = 0.045; median_TLR−2_, 2 vs. 9.2 months, *P* = 0.007; median_BTLA_, 2 vs. 8 months, *P* = 0.042; median_CTLA−4_, 2 vs. 7.7 months, *P* = 0.038; median_exosomalPD−L1_, 2 vs. 7.7 months, *P* = 0.001; Figures S2A,B). Although with no statistical difference, there was prolonged PFS in the low exosomal PD-1 and high LAG-3 expression cohort (median_exosomalPD−1_, 2 vs.4 months, *P* = 0.224, median_LAG−3_, 2 vs.7.7 months, *P* = 0.127; Figure S2B). FDR of Multiple Comparisons Correction was applied and the corrected P value was 0.011 and 0.224 for exosomal PD-L1 and exosomal PD-1 (Figure 5A). Moreover, the corrected P value of co-stimulatory and co-inhibitory factors was showed in Figure 5B (P_CD28_ = 0.026, P_CD80_ = 0.059, P_CD86_ = 0.037, P_GITRL_ = 0.037, P_ICOS_ = 0.059, P_TLR−2_ = 0.026, P_BTLA_ = 0.059, P_LAG−3_ = 0.140, P_CTLA−4_ = 0.059, Figures 5A,B).

### Multifactor Combination Detection Predicted the Response of Anti-PD-1 Therapy

Exosomal PD-L1, exosomal PD-1, CD28, CD80, CD86, GITRL, ICOS, TLR-2, BTLA, LAG-3, CTLA-4 and several clinical characteristics were included in the univariate analysis. Several potential significant indicators, including exosomal PD-L1 (HR = 3.017, *P* = 0.003), CD28 (HR = 0.394, *P* = 0.011), CD80 (HR = 0.516, *P* = 0.069), CD86 (HR = 0.409, *P* = 0.030), GITRL (HR = 0.409, *P* = 0.030), ICOS (HR = 0.494, *P* = 0.065), TLR-2 (HR = 0.348, *P* = 0.015), BTLA (HR = 0.520, *P* = 0.067), and CTLA-4 (HR = 0.511, *P* = 0.058), were selected for multivariate analysis (Table 2). Two characteristics, exosomal PD-L1 and CD28, exhibited an independent prognostic value and further validated with 1,000 times bootstrapping (HR_highexosomalPD−L1_ = 2.746, *P* = 0.009 and HR_highCD28_=0.430, *P* = 0.025, Table 2) for the PFS of patients who received anti-PD-1 therapy.

**Table 2:** Univariate and multivariate analysis for PFS in patients with anti-PD-1 therapy with 1,000 bootstraping.

	**Univariate analysis**			**Multivariate analysis**			
	**HR**	**95% CI**	***P*-value**	**HR**	**95% CI**	***P*-value**	**Bootstrapping** **95% CI**
**PFS**							
**Age**							
≤ median	1						
>median	1.167	0.577–2.357	0.668				
**Gender**							
Female	1						
Male	0.940	0.646–1.367	0.745				
**Exosomal PD-L1**							
Low	1			1			
High	3.017	1.439–6.325	0.003	2.746	1.287–5.861	0.009	1.627–8.480
**Exosomal PD-1**							
Low	1						
High	1.582	0.709–3.532	0.263				
**CD28**							
Low	1			1			
High	0.394	0.192–0.811	0.011	0.430	0.206–0.897	0.025	0.151–0.865
**CD80**							
Low	1						
High	0.516	0.253–1.054	0.069			0.723	
**CD86**							
Low	1						
High	0.409	0.182–0.915	0.030			0.789	
**GITRL**							
Low	1						
High	0.409	0.182–0.915	0.030			0.789	
**ICOS**							
Low	1						
High	0.494	0.233–1.045	0.065			0.781	
**TLR-2**							
Low	1						
High	0.348	0.149–0.812	0.015			0.386	
**BTLA**							
Low	1						
High	0.520	0.258–1.047	0.067			0.154	
**LAG-3**							
Low	1						
High	0.611	0.309–1.211	0.158				
**CTLA-4**							
Low	1						
High	0.511	0.256–1.022	0.058			0.972	
**Pathological type**							
Other	1						
Adenocarcinoma	1.106	0.568–2.153	0.768				
**Number of previous treatment lines**							
0,1	1						
≥2	1.832	0.747–4.496	0.186				
**Response of previous treatment lines**							
Non-response	1						
Response	0.865	0.392–1.910	0.720				
**Type of metastatic sites**							
Viscera organ	1						
Others	0.841	0.414–1.708	0.841				
**Number of metastatic sites**							
1	1						
≥2	1.143	0.573–2.281	0.705				

**Figure 6:**
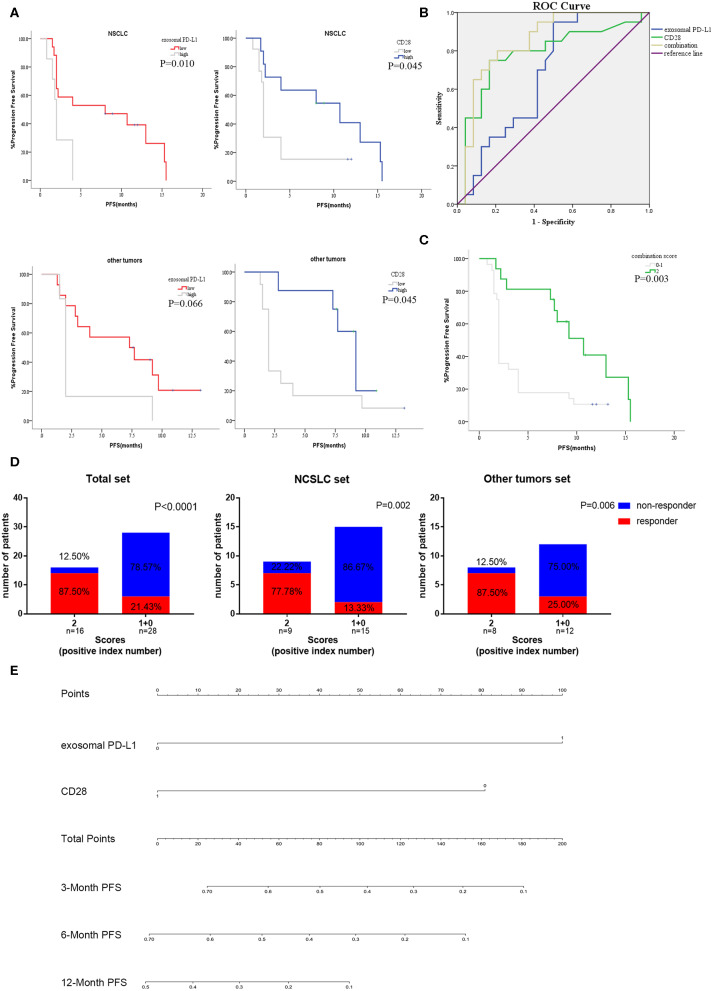
Subgroup analysis and efficiency verification of the combination of exosomal PD-L1 and CD28. **(A)** The anti-PD-1 treatment PFS of exosomal PD-L1 and CD28 in subgroup analysis [NSCLC cohort (*N* = 24) and other tumors cohort (*N* = 20)] were performed by Kaplan-Meier curves. *P*-values less than 0.05 was considered that there existed statistical differences. **(B)** Performance comparation between the two-indexes combination and single-index were illustrated by AUC of ROC. **(C)** Kaplan–Meier curves for PFS of anti-PD-1 therapy in the high scored group (two positive indexes) and low scored group (none or one positive index). *P*-values less than 0.05 was considered that there existed statistical differences. **(D)** Response rate of all patients, NSCLC set (*N* = 24) and other tumors set (*N* = 20) between high scored group (two positive indexes) and low scored group (none or one positive index) were compared by the Unpaired Student's *t*-test. *P*-values less than 0.05 was considered that there existed statistical differences. **(E)** Nomogram demonstrated the relationship between the expression of two-indexes and the PFS rate of anti-PD-1 therapy. The total points were accumulated in exosomal PD-L1 and CD28 points. The rate of 3, 6, and 12-month PFS of anti-PD-1 therapy was calculated according to the total points of patients.

Subgroup analysis was implemented to validate the applicability in different cancers. In NSCLC cohort, patients with high CD28 and low levels exosomal PD-L1 had a prolonged PFS (median_CD28_, 2 vs. 10.7 months, *P* = 0.045; median_PD−L1_, 2 vs. 8 months, *P* = 0.010; Figure 6A). Similarly, there was extended PFS in other cancer patients with high expression of CD28 or low exosomal PD-L1 value (median_CD28_, 2 vs. 9.2 months, *P* = 0.045; median _PD−L1_, 2 vs. 7.3 months, *P* = 0.066; Figure 6A).

To further confirm the prediction value of exosomal PD-L1 and CD28 expression, ROC curves were plotted to evaluate the patients without progress status. According to the ROC curve analysis, the combination of exosomal PD-L1 and CD28 exhibited more area under the curve (AUC_combination_ = 0.850, *P* < 0.001; Figure 6B) than one single biomarker (AUC_exosomalPD−L1_ = 0.678, *P* = 0.044; AUC_CD28_ = 0.784, *P* = 0.001; Figure 6B).

Therefore, we developed a two-index combination score system for the prediction of efficacy of anti-PD-1 therapy, and the scores (exosomal PD-L1 was lesser than the cutoff, and CD28 was higher than the cutoff) ranged from 0 to 2. Patients with high scores (two positive indexes, n=16) presented a longer PFS (median, 2 vs. 10.7 months, *P* = 0.003; Figure 6C) than the patients with low score (none or one positive index, *n* = 28) and were more likely to respond to anti-PD-1 therapy (87.50 vs. 21.43%, *P* < 0.0001; Figure 6D). Then, the subgroup analysis of two-index combination was performed in 24 non-small cell lung cancer (NSCLC) patients and in 20 patients with other type of tumors. Moreover, both sets suggested that the high scores cohort had a higher probability of favorable efficacy to anti-PD-1 therapy than low score cohort (NSCLC set 77.78 vs. 22.22%, *P* = 0.002; other tumors set 87.50 vs. 25.00%, *P* = 0.006; Figure 6D). A nomograph was constructed to demonstrate the correlation between the combination and progression (Figure 6E). According the analysis of the R software, the high expression of exosomal PD-L1 was assigned 100 points and the low level of CD28 was scored 80 points in the nomograph, otherwise the point was none. The total points were accumulated in exosomal PD-L1 and CD28 points. The rate of 3, 6, and 12-month PFS of anti-PD-1 therapy was calculated according to the total points of patients.

## Discussion

Based on the current study, the data revealed that exosomal PD-L1 combined with CD28 from serum could serve as an effective biomarker to predict anti-PD-1 treatment response. Before the treatment, the exosomal PD-L1 was low and CD28 expression was high in the responders. Moreover, patients with high exosomal PD-L1 and low CD28 had short PFS. Overall, the ROC curve suggested that the combined baseline exosomal PD-L1 and CD28 would be excellent indicators of predictive efficacy of PD-1 monoclonal antibody therapy.

Anti-PD-1 therapy was confirmed to be a revolutionary anti-tumor treatment in multiple tumor types with the deficiency of biomarkers. The expression of tumor PD-L1 detected by IHC was regarded as the efficacy prediction biomarker for the PD-1 monoclonal antibody. Whereas, the effect was not satisfactory due to several reasons. Firstly, the morphological heterogeneity of PD-L1, such as membrane surface PD-L1, soluble PD-L1, and exosomal PD-L1, does not completely represent the expression of PD-L1 on the membrane surface in patients (21, 22). Secondly, the spatial heterogeneity of PD-L1 and the diversified expression of PD-L1 at the various locations of tumor tissues does not represent the expression of PD-L1 of the whole tumor by IHC from a single puncture biopsy (30, 31). Finally, the temporal heterogeneity of PD-L1, wherein the expression changes at different time points, does not represent the post-treatment baseline result (30, 31). Recent studies have shown that chemotherapy drugs such as paclitaxel and cis-platinum upregulate the expression of membrane PD-L1 of tumors (32–34). In addition, the difficulty concerning specimen acquisition also casts limitations on the surface membrane PD-L1 as an effective predictor of anti-PD-1 therapeutic efficacy. In addition to the PD-L1, plenty of efficacy prediction indicators emerged, containing TMB, MSI-H/dMMR, microbiome, T cell invigoration, and gene-expression profile (GEP) (13, 35–38). However, the difficulty in obtaining specimens and the inability of continuous detection in real time limited their application and development. Unfortunately, the tumor tissues of patients in our study were rarely available and thus the TMB or MSI status was not detected. Compared with TMB, a recent research revealed that exosomal PD-L1 was more superior in terms of tumor diagnosis and prediction for ICB therapies (39). Furthermore, the priority of various detection methods was supposed to be further studied.

Several studies showed that exosomes were extracted from different patients with a variety of tumors, such as melanoma, and head and neck carcinoma. The level of exosomal PD-L1 before treatment was significantly higher in non-responders of anti-PD-1 treatment than in non-responders (22–26). Therefore, we speculated that the immunosuppressive effect of exosomal PD-L1 could lead to PD-1 antibody resistance, which was independent of the tumor type. In the current study, we have collected plasma samples from patients with advanced cancers, demonstrated the presence of exosomal PD-L1, and deduced that patients with high expression of pre-treatment exosomal PD-L1 showed a poor response to the treatment. Notably, the expression of exosomal PD-L1 was consistent with the tumor burden and efficacy evaluation. Therefore, the exosomal PD-L1 was expected to be an indicator of efficacy judgment. Otherwise, the expression of exosomal PD-L1 was not related either with membrane PD-L1 or soluble PD-L1 (22, 40, 41). Recent researches have suggested that exosomal PD-L1 was more sensitive to the membrane surface PD-L1 of melanoma patients detected by IHC (40, 41). Besides of inhibiting T cells activation and promoting tumor progression, exosomal PD-L1 could also mediate resistance to immunotherapy by directly binding to anti-PD-L1 antibody which performed different functions among other forms of PD-L1 (42). It could represent a mechanism to escape immunosurveillance and immunotherapy.

CD28, a second messenger of T cell activation, plays an indispensable role in the recognition of dendritic cells by T cells. Previous studies suggested that PD-1 antibodies rely on the activation of the CD28/B7 pathway to rescue the depletion CD8+ T lymphocytes and then achieve anti-tumor effects (43, 44). The current study has demonstrated that responsive patients with anti-PD-1 treatment had higher baseline CD28 expression than non-responsive patients, and the median PFS of the CD28 high expression group was longer than that of the low expression group. In addition, some studies reported that drugs, including chemotherapy drugs, could upregulate or degrade the expression of membrane PD-L1 of the tumors (32–34, 45), but whether the expression of exosomal PD-L1 is affected by chemotherapy or radiotherapy is not yet elucidated. Thus, to exclude this situation, we enrolled only the patients who received single-agent PD-1 monoclonal antibody. Therefore, the number of patients in this study was limited and the type of tumors was inconsistent.

Although with the limited number of patients, the current research found that the combination of exosomal PD-L1 and CD28 could be a promising predictor for response to anti-PD-1 treatment, a credible index in terms of predictive efficiency would be an effective method for screening potential population requiring the therapy. This method was employed on the patients' serum by dynamically changing the condition of the patients, which is superior to the other biomarkers. Nevertheless, we aim to increase the number of patients, validate the model in the single tumor, and verify whether various types of chemotherapeutics affect exosomal PD-L1 in future studies.

## Data Availability Statement

All datasets generated for this study are included in the article.

## Ethics Statement

The studies involving human participants were reviewed and approved by the Ethics Committee of China Medical University. The patients/participants provided their written informed consent to participate in this study.

## Author Contributions

CZ and YF performed experiments, analyzed the data, and wrote the manuscript. MZ collected clinical data. XC, CL, and SW made intellectual contributions to the method. ZL and XS made intellectual contributions to the analyses. TW and KH provided technical assistance. XQ and YL provided financial support, suggested the project and reviewed the manuscript. All authors have revised and approved the final version of the manuscript.

## Conflict of Interest

The authors declare that the research was conducted in the absence of any commercial or financial relationships that could be construed as a potential conflict of interest.

## Supplementary Material

Figure S1 Difference expression of co-inhibitory and co-stimulatory factors in responders and non-responders. The levels of four co-inhibitory factors (BTLA, TIM-3, LAG-3, and CTLA-4) and several co-stimulatory factors (CD27, CD28, CD40, HVEM, TLR-2, GITR, GITRL, ICOS, CD80, and CD86) on patients' serum were measured by the MILLIPLEX magnetic bead assay. Characterization of co-inhibitory **(A)** and co-stimulatory factors **(B)** expression in patients who responded or non-responded to PD-1 inhibitors were compared by the Unpaired Student's *t*-test. *P*-values less than 0.05 was considered that there existed statistical differences (^*^*P* < 0.05, ^**^*P* < 0.01).

Figure S2 Kaplan–Meier curves for PFS of anti-PD-1 treatment. The difference PFS of anti-PD-1 treatment between high or low group of exosomal PD-L1, exosomal PD-1, co-stimulatory factors and co-inhibitory factors were performed by Kaplan–Meier curves. **(A)** High CD28, CD80, CD86, GITRL, ICOS, TLR-2, and low exosomal PD-L1 patients showed a prolonged PFS after the anti-PD-1 treatment. The prolonged PFS in the low exosomal PD-1 patients was not statistical. **(B)** High BTLA and CTLA-4 showed a prolonged PFS. The prolonged PFS in the high LAG-3 patients was not statistical. *P*-values less than 0.05 was considered that there existed statistical differences.

## Abbreviations

AUC: area under the curve
ECOG: Eastern Cooperative Oncology Group
ELISA: enzyme-linked immunosorbent assay
HR: Hazard Ratio
IHC: immunohistochemistry
MMR: mismatch repair
MSI: Microsatellite Instability
NSCLS: Non-small cell lung cancer
PD-1: Programmed cell death 1
PD-L1: Programmed cell death Ligand 1
PFS: progression-free survival
ROC: receiver operating characteristic
TMB: tumor mutation burden
FDR: False discovery rate.
