# Supplementary figures and images for: Anti-PD-1 Therapy Response Predicted by the Combination of Exosomal PD-L1 and CD28
Source: Front Oncol. 2020 May 27;10:760. doi: 10.3389/fonc.2020.00760 (PMC7266952; doi:10.3389/fonc.2020.00760)

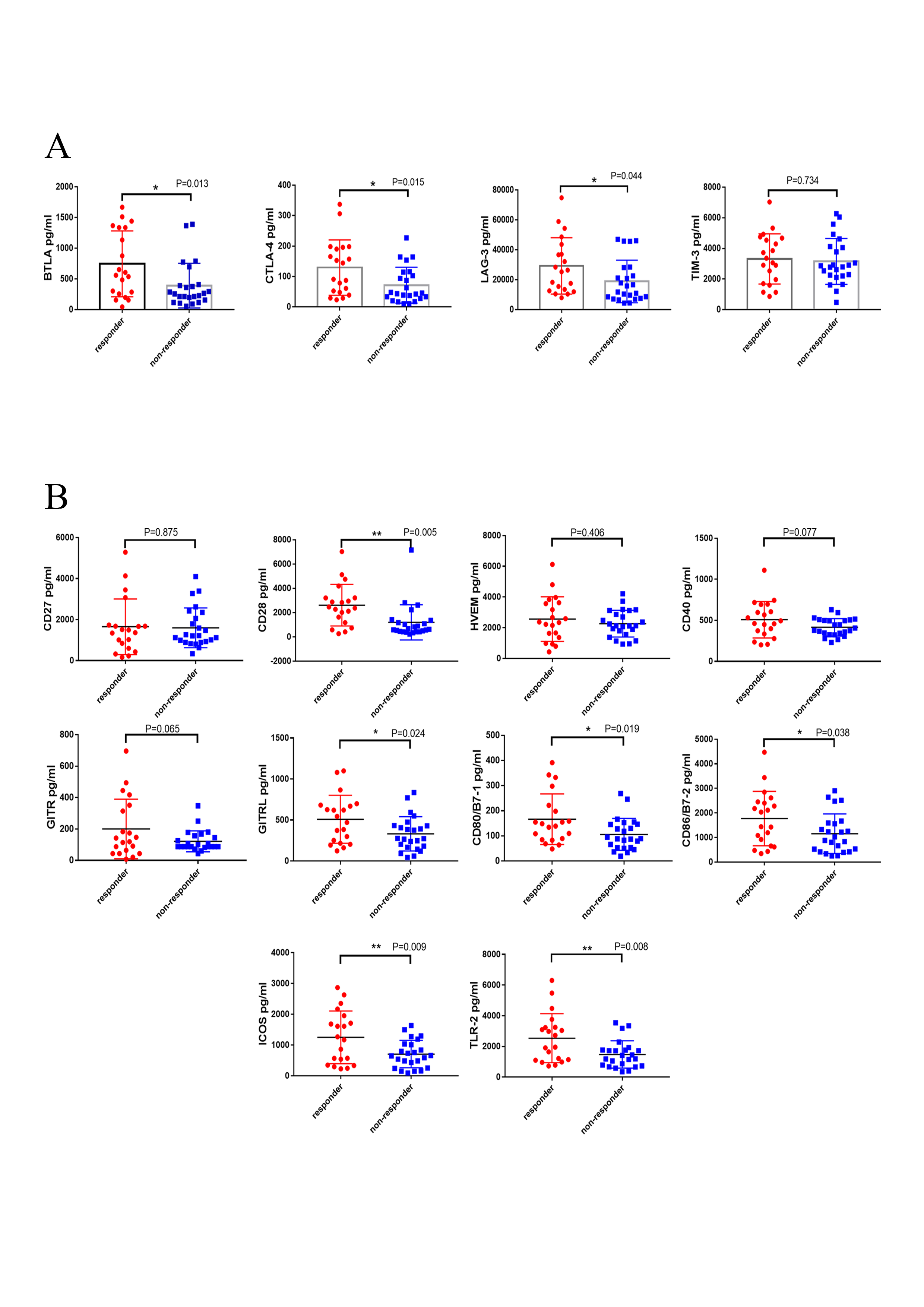

Supplement: Figure S1 — Difference expression of co-inhibitory and co-stimulatory factors in responders and non-responders. The levels of four co-inhibitory factors (BTLA, TIM-3, LAG-3, and CTLA-4) and several co-stimulatory factors (CD27, CD28, CD40, HVEM, TLR-2, GITR, GITRL, ICOS, CD80, and CD86) on patients' serum were measured by the MILLIPLEX magnetic bead assay. Characterization of co-inhibitory (A) and co-stimulatory factors (B) expression in patients who responded or non-responded to PD-1 inhibitors were compared by the Unpaired Student's t-test. P-values less than 0.05 was considered that there existed statistical differences (*P < 0.05, **P < 0.01). [file Image_1.tif]

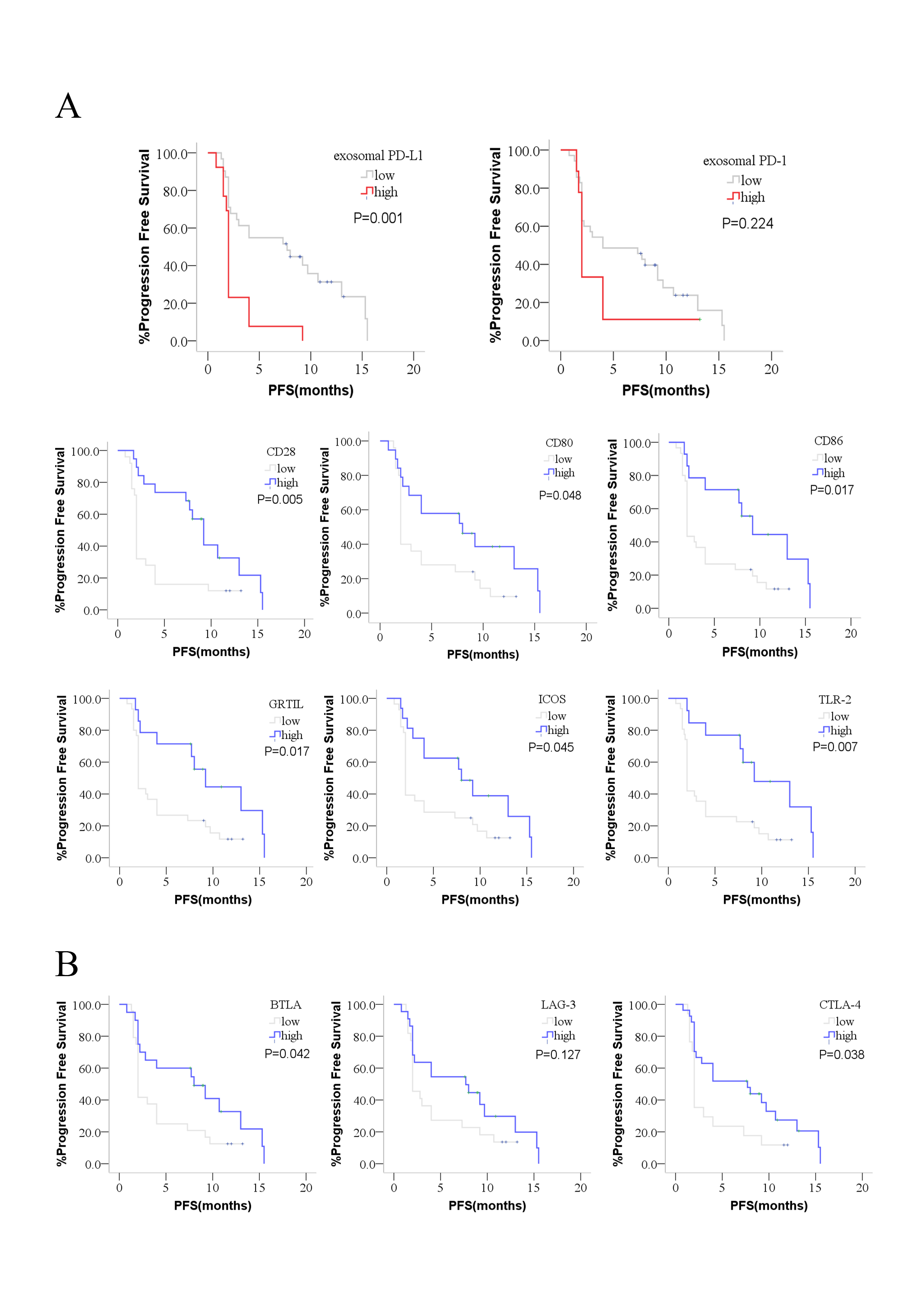

Supplement: Figure S2 — Kaplan–Meier curves for PFS of anti-PD-1 treatment. The difference PFS of anti-PD-1 treatment between high or low group of exosomal PD-L1, exosomal PD-1, co-stimulatory factors and co-inhibitory factors were performed by Kaplan–Meier curves. (A) High CD28, CD80, CD86, GITRL, ICOS, TLR-2, and low exosomal PD-L1 patients showed a prolonged PFS after the anti-PD-1 treatment. The prolonged PFS in the low exosomal PD-1 patients was not statistical. (B) High BTLA and CTLA-4 showed a prolonged PFS. The prolonged PFS in the high LAG-3 patients was not statistical. P-values less than 0.05 was considered that there existed statistical differences. [file Image_2.tif]
